# Effects of Positive Language and Profession on Trustworthiness and Credibility in Online Health Advice: Experimental Study

**DOI:** 10.2196/16685

**Published:** 2020-03-10

**Authors:** Lars König, Regina Jucks

**Affiliations:** 1 Department of Psychology University of Münster Münster Germany

**Keywords:** health communication, information-seeking behavior, language, occupations, trust

## Abstract

**Background:**

When searching for health information, many people use the internet as their first source of information. In online health forums, for example, users post their questions and exchange health advice. In recent years, information givers from various professions have begun to use positive language (indicated by the frequent use of positively valenced adjectives) to communicate their information and persuade their audiences.

**Objective:**

The goal of the current study was to answer the following research questions: (1) How does positive language, in comparison to neutral language, influence the trustworthiness of a person arguing in an online health forum and the credibility of their health claims; (2) How does working for a university, compared to working for a lobbying organization, influence the trustworthiness of a person arguing in an online health forum and the credibility of their health claims; and (3) Do the two factors of language style and professional affiliation interact with each other to influence trustworthiness and credibility judgments?

**Methods:**

In a 2 × 2 between-subject experiment, 242 participants read a post from an online health forum and subsequently rated the trustworthiness of the forum post author and the credibility of their information. Within the post, the professional affiliation (scientist vs lobbyist) and language style (neutral vs positive) of the forum post author was varied.

**Results:**

When the forum post author used a positive language style, they were perceived as less trustworthy (high Machiavellianism [*P*<.001; η^2^_p_=.076], low Integrity [*P*=.001; η^2^_p_=.045], and low Benevolence [*P*=.02; η^2^_p_=.025]) and their information was perceived as less credible (low Message Credibility [*P*=.001; η^2^_p_=.045]). The professional affiliation of the forum post author did not influence their trustworthiness or the credibility of their information.

**Conclusions:**

When searching for health information, information seekers evaluate the language style of forum posts to decide whether forum post authors are trustworthy and their information is credible.

## Introduction

### Evaluating the Validity of Online Health Information

When searching for health information, many people use the internet as their first source of information [1,2]. In online health forums, for example, users write about their symptoms and get treatment advice from other users [3]. Furthermore, online health forums can provide patients with emotional as well as informational support [4]. However, online health information is not always accurate and often contains misinformation [5-8]. Thus, health information seekers must decide whether they should rely on health claims they encounter online. Based on the reasoning of the content-source integration model [9,10], one strategy to make such decisions is to evaluate the credibility of the provided information (eg, “Is the health claim logical, coherent and compatible with my prior knowledge?”) and the trustworthiness of the information source (eg, “Are there any reasons why the information source might lie?”). Since health information claims can be highly complex, and most people have just a limited understanding of science [11,12], it is often difficult for information seekers to evaluate health claims accurately. In such situations, information seekers base their credibility and trustworthiness judgments on factors that surround health claims. Two factors that seem especially important are the professional affiliation of an information source and the information source’s language style [13-23].

For example, one study showed that unfamiliar health information is rated as being more credible if the information source has a professional affiliation indicating they are a medical expert (eg, Dr. William Blake, HIV specialist) rather than a nonexpert (eg, Tim Alster, a high school freshman) [22]. Another study found that health experts are perceived as less trustworthy and their information as less credible if their professional affiliation suggests a potential conflict of interest (eg, experts working for organic food lobbying organizations who argue that organic food is superior to conventional food and base their argumentation on studies that they have conducted themselves) [21]. In the context of language styles, it has been shown that information sources who aggressively communicate health information are judged to be less trustworthy, and their information is deemed less credible [19]. Aggressive language, however, is not the only language style that influences trustworthiness and credibility ratings: enthusiastic [20], technical [17], tentative [14], and conversational [23] language styles also influence the credibility of online health information and the trustworthiness of information sources. Furthermore, it has been shown that language and word choices do not just influence credibility and trustworthiness judgments, but other outcomes as well [24-26]. For example, medical students answer emotional patient queries more emotionally [24], and the use of narratives can influence risk perceptions [25].

### How Does Positive Language Influence the Credibility of Health Claims and the Trustworthiness of Health Communicators?

Using positive language, indicated by the implementation of positively valenced adjectives (eg, great, amazing, outstanding), is another language style that seems to be on the rise when it comes to communicating health and science information. For example, in an article for the National Academy of Sciences, the authors did not just neutrally write about an antioxidant. Instead, they chose to write about an “outstanding antioxidant” [27]. Furthermore, in more recent scientific articles, interested readers can learn about “fascinating fasciclins” [28] and “the amazing world of bacteriophage diversity” [29]. These are just three of many examples from scientists who use positive language to communicate their findings. Interestingly, when turning to the popular press, authors seem to become even more eager to use positive language to communicate their advice. Here, health information seekers can read about “amazing diet recipes for weight loss” [30], “genius health tips from around the world” [31], and “an incredible therapy for modern day conditions” [32].

Authors who use such positive language might want to stress the importance of their advice or the quality of their research. However, is this a reasonable strategy in the context of health communication? Moreover, how does positive language influence the trustworthiness of health communicators and the credibility of their health claims? To our knowledge, no research has addressed these questions so far. However, it is known from previous research that businesses often use positive language to generate a favorable view of their prospects and performances [33-35], and this technique seems to be effective in some circumstances. In one study, for example, participants saw hard-to-understand financial disclosure statements that were written in either a positive or a neutral language style [34]. If the participants had little financial knowledge and saw the positively written statement, they indeed thought that the company in question would have higher earnings in the future. Other research that explored the effects of enthusiastic language has shown that expressing too much enthusiasm about a topic (eg, “And what I can tell you at the beginning: I think the topic is fascinating!”) might backfire and decrease credibility and trustworthiness [20]. However, another study found that listening to an enthusiastic version of a podcast, in comparison to a neutral version of the same podcast, resulted in more positive instructional quality ratings: Participants who listened to the enthusiastic version rated it as more interesting, and they perceived the podcast host as more trustworthy [36]. Furthermore, in line with language expectancy theory [37], the credibility of an information source might moderate whether using a positive language style is appropriate or not: High-credibility sources like scientists might have the freedom to choose between different language styles without putting their trustworthiness at risk. Low-credibility sources like lobbyists, on the other hand, might not have the same freedom of choice when it comes to language styles.

Hence, diverse effects of positive language seem to be possible. If an information source uses positive language when writing about the effectiveness of a specific drug, information seekers can interpret this language choice in different ways. On the one hand, information seekers might conclude that the positive language style shows that the information source is highly convinced of the effectiveness of the drug and wants to express their excitement. In this case, the positive language style might function as a quality cue that increases credibility and trustworthiness. On the other hand, the positive language style might remind information seekers of commercials that are designed to persuade their audiences and increase sales. In this case, the positive language style might function as a negative cue that decreases credibility and trustworthiness. Furthermore, one might argue that positive language is more likely to be perceived as a quality cue if a scientist uses it because scientists are typically not interested in increasing the sales of a specific drug. However, one could also argue that people expect a scientist to be neutral and objective, and therefore the use of positive language will be perceived as a negative cue.

This example and the previously discussed literature illustrate two things: (1) many authors with different professional affiliations use positive language, indicated by the implementation of positively valenced adjectives, to communicate health information; and (2) little is known about how such positive language influences the trustworthiness of health communicators and the credibility of their health claims. Therefore, we designed an experiment to investigate how different language styles and different professional affiliations influence the trustworthiness of health communicators and the credibility of their health claims. During the experiment, participants read a post from an online health forum and subsequently rated the trustworthiness of the forum post author and the credibility of their information. Within the forum post, we varied the professional affiliation of the forum post author (whether the person was a scientist or a lobbyist) and their language style (whether they used neutral or positive language). Because the previously discussed research has shown that positive language can have either positive or negative effects, no directional hypotheses were stated. Instead, the goal was to answer the following nondirectional research questions: (1) How does positive language, in comparison to neutral language, influence the trustworthiness of a person arguing in an online health forum and the credibility of their health claims; (2) How does working for a university, in comparison to working for a lobbying organization, influence the trustworthiness of a person arguing in an online health forum and the credibility of their health claims; and (3) Do the two factors of language style and professional affiliation interact with each other to influence trustworthiness and credibility judgments?

## Methods

### Design and Material

A 2 (language style: neutral language vs positive language) × 2 (professional affiliation: scientist vs lobbyist) between-subject experimental design was used, resulting in four experimental conditions. In each experimental condition, participants saw two online forum posts: a question post and an answer post. In the question post, a woman asked whether Batradicum was an effective drug for the treatment of attention deficit hyperactivity disorder (ADHD). The question post was written in a neutral language style and was the same in all four experimental conditions. In the answer post, a man introduced himself and argued that Batradicum is an effective drug for the treatment of ADHD. The experimental manipulations were realized in the answer post. Depending on the experimental condition, the answer post was written either in a neutral language style or a positive language style. Furthermore, the author of the answer post introduced himself either as a scientist who worked for a pharmacological institute at a university, or as a lobbyist who worked at a pharmacological lobbying organization. Textbox 1 shows the question post and the answer post with the experimental manipulation.

As a note, the positive language style version of the answer post contained the words and phrases printed in italics, and the neutral language style version did not contain these words and phrases. For reasons of ecological validity, the name of an existing ADHD medication was used in the original study material. To avoid the impression that the authors endorse or criticize the drug, the fictitious drug name Batradicum is used here. Also, as the table shows an English translation of the German posts, the translated version may not appear as authentic to native English speakers as the original version appears to native German speakers. The original German version of the posts can be obtained from the authors upon request.

**Text of the question and answer post.**

**Question post:**

Dear forum community,

My son is, according to his teachers, hyperactive and cannot concentrate in class. Moreover, his grades are suffering and he might not be allowed to move up to the next grade. The doctor gave him an ADHD diagnosis and offered that he could prescribe him the drug Batradicum. Unfortunately, I am not an expert in this field and I have heard of different studies that either argue for or against the effectiveness of Batradicum.

Does anybody know more about this topic?

Thanks in advance!

Sabine Schneider

**Answer post:**

Hello Ms. Schneider,

My name is Johannes Becker and since I have been working for the [Scientist Manipulation: *Pharmacological Institute at the University of Bochum*; Lobbyist Manipulation: *Association of Pharmacological Industries in Bochum*] for many years, I have been dealing with the subject of Batradicum for quite some time.

You are right, in the past, many studies on Batradicum have contradicted each other, often because of methodological mistakes.

Recently, however, a *magnificent* study by Mr. Weber has been published, which speaks for the effectiveness of Batradicum. Mr. Weber has *brilliantly* compared different age groups, which many previous studies have not done. In addition, in his *unique* study, he has studied not only the physiological but also the psychological effects of Batradicum, which is rare *and especially praiseworthy*. His *exemplary* methodological approach and *first-class* statistical data analysis speak for the quality of the study. Due to the *really outstanding* study of Mr. Weber and its *excellent* execution, I am convinced that Batradicum is effective.

Yours sincerely,

Johannes Becker

### Sample and Procedure

German university students were contacted via email and social network sites and received €5 (US $5.65) for participating in the experiment. Overall, 251 participants completed the study without interruption, but 7 participants were excluded from data analysis because they indicated that they encountered technical problems during the study. Furthermore, 2 participants were excluded from data analysis because they indicated that they did not answer the questions honestly. The final sample contained 242 (165 females, 77 males) students (175 undergraduate students, 67 graduate students) at an average age of 23 years old (mean 22.57; SD 3.05). The average participant had been enrolled in their study program for five semesters (mean 4.81; SD 3.38). The experiment was conducted online using the EFS Survey platform (Questback GmbH, Cologne, Germany). First, participants gave informed consent, provided demographic information, and answered the control measures. They were then randomly assigned to one of the four experimental conditions. After reading the forum posts, participants answered the dependent measures and the manipulation check question. At the end of the experiment, the participants were debriefed.

### Control Measures and Manipulation Check

A total of four control measures were included to assess whether the experimental groups differed in characteristics that could bias the study results. Participants answered three questions [General Use: “How often do you visit Internet forums?”; Educational Use: “How often do you visit Internet forums to learn something new or acquire new skills?”; Prior Knowledge: “How much do you know about Batradicum?”) and indicated their agreement with one statement (Prior Attitude: “Batradicum is an effective drug for the treatment of ADHD.”) on seven-point scales. To check whether the language style manipulation was successful, participants answered the question, “How would you describe Johannes Becker’s choice of words?” on a scale ranging from 1 (neutral) to 7 (extremely enthusiastic).

### Dependent Measures

To assess the credibility of the provided information, two measures were used, one of which was a general credibility measure, the Message Credibility Scale [38]. This measure was translated and adapted. As a more specific credibility measure, participants were also asked how much they agreed with the main conclusion of the forum post author (Attitude). To assess the trustworthiness of the information source, five measures were used: the German version of the Machiavellianism Subscale from the Dirty Dozen Scale [39,40] was used to assess how manipulative the forum post author was perceived to be (Machiavellianism), the Muenster Epistemic Trustworthiness Inventory [41] was used to assess how knowledgeable (Expertise), sincere (Integrity), and benevolent (Benevolence) the forum post author was perceived to be, and as a more general trustworthiness measure, the Reysen Likability Scale [42] was used to assess how likable the forum post author was perceived to be (Likability). Participants gave their answers on 7-point scales. The original dataset contains further variables that have not been described because they exceed the scope of the present article.

## Results

### Control Measures and Manipulation Check

For all analyses, the statistical software SPSS Statistics Version 26 (IBM Corp, Armonk, New York, United States) was used. Four one-way between-subject analyses of variance were conducted with experimental condition as the independent variable and the control measures as dependent variables. The results showed that the participants in the four experimental groups did not significantly differ in regard to the control measures of General Use (*F*_3,238_=1.339; *P*=.262), Educational Use (*F*_3,238_=0.784; *P*=.504), Prior Knowledge (*F*_3,238_=0.505; *P*=.679), and Prior Attitude (*F*_3,238_=1.365; *P*=.254). Furthermore, participants in the positive language style condition (mean 5.97; SD 1.28) perceived the choice of words as more enthusiastic than participants in the neutral language style condition (mean 3.63; SD 1.67; tt_227.769_=–12.231; *P*<.001). Thus, the language style manipulation worked as expected.

### Dependent Measures

For the analyses of the dependent measures, two-way between-subject analyses of variance were conducted with language style (neutral language vs positive language) and professional affiliation (scientist vs lobbyist) as independent variables.

#### Main Effects of Language Style

There were significant main effects of language style on Message Credibility (*F*_1,238_=11.274; *P*=.001; η^2^_p_=.045), Machiavellianism (*F*_1,238_=19.621; *P*<.001; η^2^_p_=.076), Integrity (*F*_1,238_=11.328; *P*=.001; η^2^_p_=.045), and Benevolence (*F*_1,238_=6.036; *P*=.02; η^2^_p_=.025). However, there were no main effects of language style on Attitude (*F*_1,238_=0.785; *P*=.38; η^2^_p_=.003], Expertise (*F*_1,238_=2.045; *P*=.15; η^2^_p_=.009], and Likability (*F*_1,238_=1.721; *P*=.19; η^2^_p_=.007).

#### Main Effects of Professional Affiliation

There were no main effects of professional affiliation on the dependent measures of Message Credibility (*F*_1,238_=2.852; *P*=.09; η^2^_p_=.012), Attitude (*F*_1,238_=1.122; *P*=.29; η^2^_p_=.005), Machiavellianism (*F*_1,238_=1.144; *P*=.29; η^2^_p_=.005), Expertise (*F*_1,238_=0.400; *P*=.53; η^2^_p_=.002), Integrity (*F*_1,238_=1.401; *P*=.24; η^2^_p_=.006), Benevolence (*F*_1,238_=0.429; *P*=.51; η^2^_p_=.002), and Likability (*F*_1,238_=2.594; *P*=.11; η^2^_p_=.011).

#### Interaction Effects

**Figure 1:**
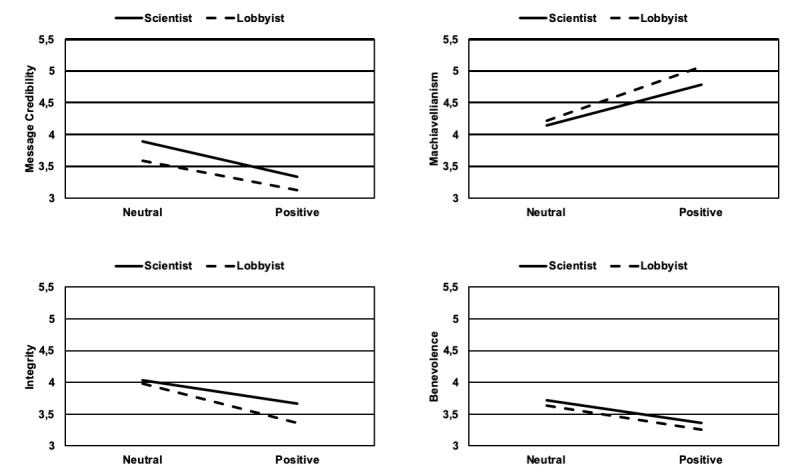
Dependent measures that were significantly influenced by language style.

The two factors of language style and professional affiliation did not interact with each other to influence trustworthiness and credibility judgements for Message Credibility (*F*_1,238_= 0.091; *P*=.76; η^2^_p_<.001), Attitude (*F*_1,238_=0.112; *P*=.74; η^2^_p_<.001), Machiavellianism (*F*_1,238_=0.397; *P*=.53; η^2^_p_=.002), Expertise (*F*_1,238_=0.559; *P*=.46; η^2^_p_=.002), Integrity (*F*_1,238_=0.725; *P*=.40; η^2^_p_=.003), Benevolence (*F*_1,238_=0.007; *P*=.93; η^2^_p_<.001), and Likability (*F*_1,238_=3.708; *P*=.06; η^2^_p_=.015). Table 1 shows the means and standard deviations of the dependent measures by language style and professional affiliation. Figure 1 shows the dependent measures that were significantly influenced by the language style manipulation.

As a note for Table 1, for the Machiavellianism scale, a low score indicates high trustworthiness and a high score indicates low trustworthiness. For all other scales, a low score indicates low trustworthiness/credibility and a high score indicates high trustworthiness/credibility. All scales ranged from 1-7.

**Table 1:** Main effects of the dependent measures by language style and professional affiliation.

Dependent measures			Language style					Professional affiliation					
			Neutral (n=123), mean (SD)		Positive (n=119), mean (SD)		*P* value		Scientist (n=124), mean (SD)		Lobbyist (n=118), mean (SD)		*P* value
**Credibility**													
	Message Credibility	3.75 (1.19)		3.24 (1.20)		.001		3.62 (1.25)		3.36 (1.17)		.09	
	Attitude	3.80 (1.42)		3.95 (1.22)		.38		3.96 (1.29)		3.78 (1.36)		.29	
**Trustworthiness**													
	Machiavellianism	4.18 (1.32)		4.93 (1.29)		<.001		4.46 (1.46)		4.64 (1.24)		.29	
	Expertise	4.45 (1.20)		4.23 (1.14)		.15		4.39 (1.23)		4.29 (1.12)		.53	
	Integrity	4.00 (1.17)		3.51 (1.10)		.001		3.85 (1.14)		3.68 (1.19)		.24	
	Benevolence	3.68 (1.20)		3.31 (1.11)		.02		3.54 (1.20)		3.45 (1.13)		.51	
	Likability	3.37 (0.97)		3.22 (0.97)		.19		3.39 (0.96)		3.19 (0.97)		.11	

## Discussion

### Discussion of the Research Questions

The goal of the present study was to explore whether the language style (positive vs neutral) and professional affiliation (scientist vs lobbyist) of a person communicating health information in an online forum would influence their trustworthiness and the credibility of their health claims. The results show that if the forum post author used positive language in comparison to neutral language, they were perceived as less trustworthy, and their health claims were deemed less credible. More specifically, if the forum post author used positive language, they were perceived as more manipulative (Machiavellianism), less sincere (Integrity), less benevolent (Benevolence), and their health claims were perceived as less credible (Message Credibility).

In contrast, the professional affiliation of the forum post author (whether they were a scientist or a lobbyist), affected neither their trustworthiness nor the credibility of their information. This result is surprising because it contradicts previous findings [19]. On closer examination, however, the descriptive statistics show that the lobbyist was rated as less trustworthy on every measure, even though these differences did not reach significance. Therefore, one might speculate that no effect of professional affiliation was found because the experimental manipulation was too weak (just one sentence at the beginning of the forum post), or the participants did not identify the Association of Pharmacological Industries in Bochum as a lobbying organization. Lastly, the language style and professional affiliation manipulations did not interact with each other.

### Limitations and Future Research Directions

Even though the results of the current study increase the understanding of how information seekers assess the accuracy of health information in online forums, there are limitations to the generalizability of the results. Four limitations seem especially important. First, the study participants were relatively young due to their status as students. Since previous research has found that age might influence source monitoring, suggestibility to misinformation [43], and credibility judgments in online contexts [44], future research should replicate the current study with different age groups. Second, the language style manipulation was realized by incorporating multiple positively valenced adjectives in the forum post. Since the number of adjectives has presumably influenced the language style effect, future research should replicate the current study with varying amounts of adjectives to explore the boundary conditions of the found effect. This suggestion seems especially important because different amounts of adjectives might alter the direction of the language style effect. For example, if an information source uses numerous positive adjectives in a health forum, information seekers might perceive this language use as inappropriate because it reminds them of advertising campaigns that often use extremely positive language. However, if an information source uses just a few positive adjectives, information seekers might perceive this language use as typical for a person who sincerely believes in their position. Third, the manipulation check showed that participants in the positive language style condition perceived the choice of words as more enthusiastic than participants in the neutral language style condition. However, the neutral language style was not perceived as entirely neutral. Instead, it was perceived as moderately enthusiastic. One might argue that the current results, therefore, represent differences between moderately enthusiastic and extremely enthusiastic language styles. Hence, future research should replicate the current study with new experimental material that is perceived as more neutral. Fourth, the current study employed a highly specific health-seeking context: A female information seeker asked for health advice regarding a specific drug and got an answer from a male information source. This specific context might have influenced the results. Previous research has shown that many members of the public have negative attitudes towards the use of drugs to treat ADHD [45]. Therefore, arguing in favor of using drugs to treat ADHD might have intensified the negative effects of the positive language style manipulation.

Consequently, future research should explore the positive language style effect within the context of less controversial topics. Furthermore, previous research suggests that the impact of expert testimony is influenced by the congruency between the gender of the expert and the topic at hand [46]. Thus, it would be interesting to manipulate the gender of the information source and investigate whether this alters the effect of the positive language style manipulation. Furthermore, it would be interesting to investigate whether the gender of the information source and the gender of the information seeker interact with each other when influencing credibility and trustworthiness judgments.

### Conclusion

When searching for health information, many people use the internet as their first source of information. When they are confronted with positive language in online health forums, indicated by the frequent use of positively valenced adjectives, they may judge the health communicator as less trustworthy and deem the communicated health claims as less credible. These findings illustrate that health information seekers do not just react to the factual part of health information. Instead, they use the language style that surrounds health claims to evaluate the credibility of the provided information and the trustworthiness of the information source.

## Abbreviations

ADHD: attention deficit hyperactivity disorder
